# Efficacy of progressive versus severe energy restriction on body composition and strength in concurrent trained women

**DOI:** 10.1007/s00421-023-05158-8

**Published:** 2023-02-17

**Authors:** Salvador Vargas-Molina, Diego A. Bonilla, Jorge L. Petro, Leandro Carbone, Manuel García-Sillero, José Manuel Jurado-Castro, Brad J. Schoenfeld, Javier Benítez-Porres

**Affiliations:** 1 Physical Education and Sports Area, Faculty of Medicine, University of Málaga, Bulevar Louis Pasteur, 25, 29010 Málaga, Spain; 2 EADE-University of Wales Trinity Saint David, Málaga, Spain; 3 Research Division, Dynamical Business and Science Society–DBSS International SAS, Bogotá, Colombia; 4 Research Group in Physical Activity, Sports and Health Sciences, Universidad de Córdoba, Montería, Colombia; 5 Sport Genomics Research Group, Department of Genetics, Physical Anthropology and Animal Physiology, Faculty of Science and Technology, University of the Basque Country (UPV/EHU), 48940 Leioa, Spain; 6 University of Salvador, Buenos Aires, Argentina; 7 Metabolism and Investigation Unit, Maimonides Biomedical Research Institute of Cordoba (IMIBIC), Reina Sofia University Hospital, University of Cordoba, 14004 Córdoba, Spain; 8 Department of Health Sciences, CUNY Lehman College, New York, USA; 9 CIBERobn Physiopathology of Obesity and Nutrition, Centre of Biomedical Research Network, Institute of Health Carlos III, 28029 Madrid, Spain; 10 Osuna University School, Teaching Center Attached to the University of Seville, 41640 Seville, Spain

**Keywords:** Concurrent training, Calorie restricted diet, Fat reduction, Hypertrophy, Muscle strength, Resistance training

## Abstract

**Purpose:**

This study evaluated the concurrent training (CT) effect in combination with either progressive energy restriction (PER) or severe energy restriction (SER) on body composition and strength-related variables in resistance-trained women.

**Methods:**

Fourteen women (29.5 ± 3.8 years; 23.8 ± 2.8 kg·m^−2^) were randomly assigned to a PER (*n* = 7) or SER (*n* = 7) group. Participants performed an 8-week CT program. Pre- and post-intervention measures of fat mass (FM) and fat-free mass (FFM) were assessed by dual-energy X-ray absorptiometry and strength-related variables were assessed through 1-repetition maximum (in the squat and bench press) and countermovement jump.

**Results:**

Significant reductions in FM were observed in PER and SER (Δ = − 1.7 ± 0.4 kg; *P* =  < 0.001; ES = − 0.39 and Δ = − 1.2 ± 0.6 kg; *P* = 0.002; ES = − 0.20, respectively). After correcting FFM for fat-free adipose tissue (FFAT), no significant differences for this variable were found either in PER (Δ = − 0.3 ± 0.1; *P* = 0.071; ES = − 0.06) or in SER (Δ = − 0.2 ± 0.1; *P* = 0.578; ES = − 0.04). There were no significant changes in the strength-related variables. No between-group differences were found in any of the variables.

**Conclusion:**

A PER has similar effects to a SER on body composition and strength in resistance-trained women performing a CT program. Given that PER is more flexible and thus may enhance dietary adherence, it might be a better alternative for FM reduction compared to SER.

## Introduction

Severe energy restriction (SER) strategies have been used for years for the purpose of reducing fat mass (FM) levels. SER is often combined with regimented fitness programs to enhance fat loss while preserving fat-free mass (FFM). Alternatively, competitive fitness and bodybuilding competitors often employ progressive energy restriction (PER) strategies of durations between two and four months, in conjunction with resistance training and often cardiovascular exercise (a.k.a., concurrent training) (Maestu et al. 2010). The duration of these restrictions depends on the athlete’s physical fitness, the time remaining for the competition, and the individual genetic characteristics of the athlete. Regardless, a reduction in FM requires an energy deficit (McGuire 2011) and/ or an increase in total daily energy expenditure through physical exercise (Deighton et al. 2014); both of which can compromise fat free mass (FFM) levels. Higher energy deficits are associated with more rapid weight loss; the more pronounced the deficit, the greater the possibility to lose lean mass (Hall 2008; Mettler et al. 2010; Garthe et al. 2011). Moreover, evidence shows that a prolonged energy deficit in natural bodybuilders could also lead to hormonal imbalances, fatigue and psychological issues (Fagerberg 2018).

To counteract the detrimental effects of a negative energy balance, fat loss interventions should seek to employ strategies that maintain as much FFM as possible (Helms et al. 2014). For example, research indicates that higher intakes of dietary protein aid in the preservation of FFM during periods of energy restriction (Wycherley et al. 2012; Walilko et al. 2021). For trained athletes under hypocaloric conditions, recommendations range from 1.8 to 2.7 g/protein/kg/day (Helms et al. 2014), and 1.6–2.4 g/protein/kg/day (Hector and Phillips 2018). Moreover, resistance training helps to enhance the maintenance of FFM during an energy deficit (Josse et al. 2011; Longland et al. 2016). Despite the efficacy of these strategies, the loss of FFM is not always attenuated during SER (Gornall and Villani 1996; Donnelly et al. 1991). For example, the only study to date that evaluated the effects of SER with a high protein diet on body composition and physical performance (including muscular strength) in well-trained female athletes of different disciplines (Pearson et al. 2021) found no differences in FFM between groups consuming a high (35% of energy intake) or modest (15% of energy intake) protein intake.

Due to a paucity of research in female athletes as to the effects of energy restriction combined with regimented exercise, the aim of this study was to compare the effects of an 8-week high-protein SER or PER dietary intervention accompanied by a high-volume concurrent training (CT) program on body composition and strength-related variables in resistance-trained women. We hypothesized that eight weeks of a PER intervention would elicit a greater reduction in FM while maintaining FFM and strength levels compared to a SER due to a better adherence to the nutritional strategy in resistance-trained women.

## Materials and methods

### Experimental design

This was a repeated-measures study carried out in resistance-trained women who were recruited from a previous research project (Romance et al. 2019; Vargas-Molina et al. 2020) to participate immediately following completion of that protocol. After previously taking part in an 8-week training and nutritional protocol with a consumption of 45 kcal·kg^−1^ FFM, the participants were randomly assigned in a 1:1 fashion to either a SER or PER dietary intervention accompanied by a high-volume CT program. This design allowed the athletes to become adapted to the program protocols and thus help to ensure better internal validity.

### Sample

Fourteen young women (29.5 ± 3.8 years; 164.9 ± 7.0 cm; 63.1 ± 9.0 kg; 23.8 ± 2.8 kg·m^−2^) with > 2 years of continuous experience in resistance training volunteered to participate in this study. Participants who self-reported the use of doping agents (e.g., anabolic–androgenic steroids) during the last two years were excluded from participation. Additionally, participants pledged not to consume ergogenic supplements during the study. Female athletes with oligomenorrhea or polycystic ovarian syndrome, as well as those not within the required age range of 18–35 years, were excluded as well.

All participants committed to following the CT and dietary protocols, and to be monitored during the 8-week study period. Participants were instructed to avoid performing any structured exercise during the study period other than that prescribed for the intervention. The participants were informed of the possible risks of the experiment and signed an informed consent form. The study was developed in accordance with the ethical guidelines of the World Medical Association Declaration of Helsinki (WMA 2013) and approved by the ethics committee at University of Málaga (code: 38-2019-H).

### Exercise protocol

**Fig. 1:**
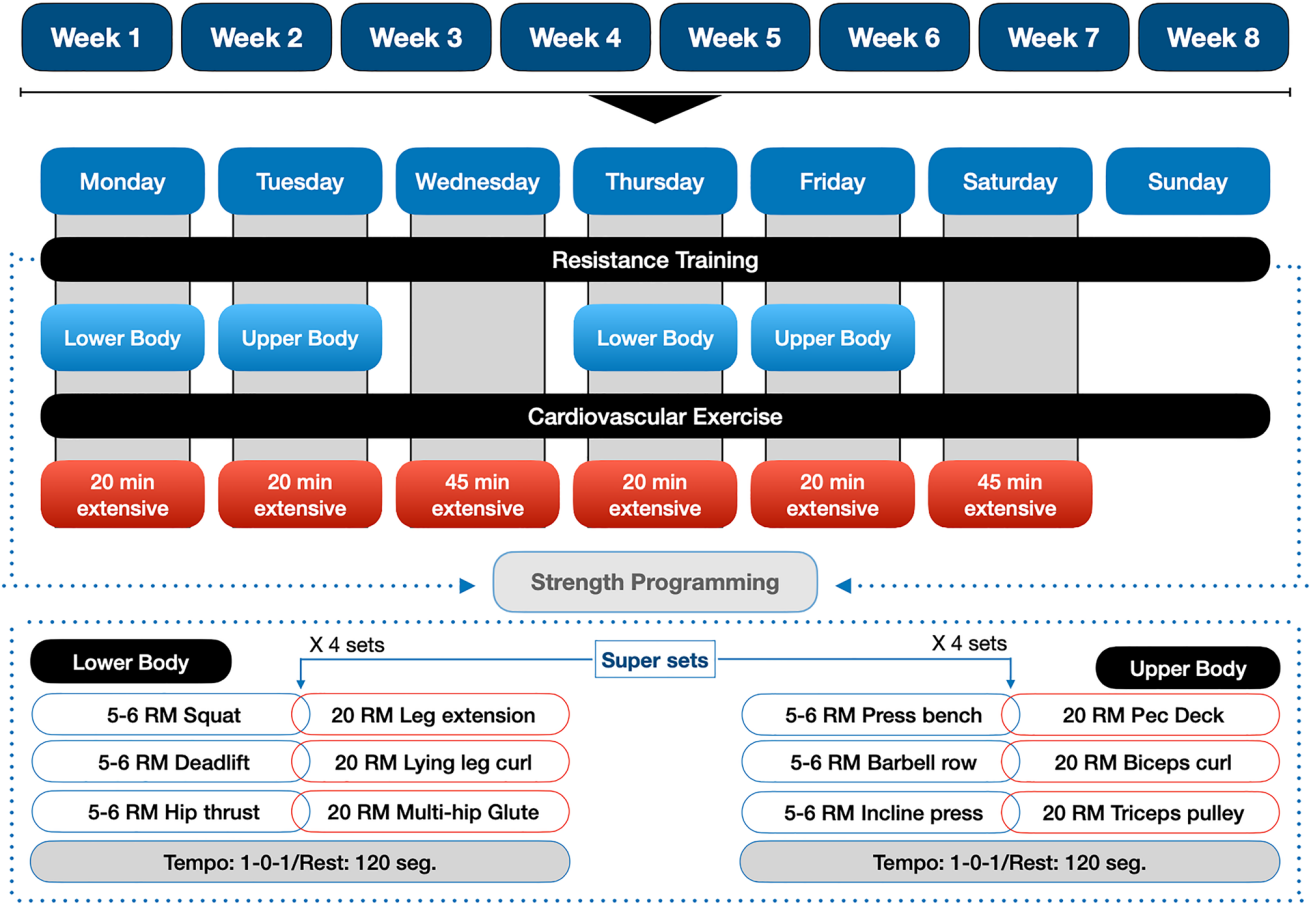
Organization of the resistance and cardiovascular training protocol. 1–0–1 = a second eccentric phase, zero isometric and one second in the concentric. RM repetition maximum

Both groups performed a CT program with a frequency of 6 sessions a week. The resistance training program consisted of four sessions a week, divided into upper-limb/lower-limb, with a 72-h recovery period between sessions that involved training the same muscle region. The program incorporated super-sets (two exercises are performed in succession), whereby the first exercise, a multi-joint movement, was performed with relatively heavy loads (~ 5–6 repetition maximum [RM]) and the second exercise, a single-joint movement, was performed with lighter loads (~ 20RM). A 3-min rest interval was implemented between each super-set. Participants trained to volitional failure during the first 3 weeks of the study, and in the fourth week the loads were decreased; this sequence was replicated over the ensuing 4 weeks of the study. At the end of the resistance training sessions, participants performed 20 min of cardiovascular exercise on a cycle ergometer at an intensity of 65% heart rate reserve. Additionally, the participants carried out two 45-min cardio sessions on non-resistance training days. Thus, the total weekly volume of cardiovascular exercise was 170 min, with the last day of the week allocated for recovery. The intensity of the cardiovascular exercise was controlled using the estimation of the heart rate reserve via the Karvonen method (Karvonen et al. 1957). The following formula was used. To calculate maximum heart rate: 206 − (0.88 × age) (Gulati et al. 2010) and apply it to the Karvonen formula. Participants were asked to record resting heart rate for three consecutive mornings (the mean of the records was used) immediately upon waking and before arising. Participants used heart rate sensor to assess heart rate (Sensors Polar H10, Tampere, Finland), wetting the band as indicated by the manufacturer. The programming variables, intensity, rest and cadences were adjusted to each type of training (Fig. 1). All participants performed the same exercise protocol for the duration of the program. During the intervention, all participants were monitored by a physical conditioning specialist, who supervised, controlled and adjusted the loads in each training session.

### Dietary intervention

The intervention began immediately after participants completed an 8-week program designed to increase FFM with a daily intake of 45 kcal·kg^−1^ FFM (Romance et al. 2019; Vargas-Molina et al. 2020). The SER group was instructed to consume 25 kcal·kg^−1^ FFM from throughout the 8-week duration of the present investigation. The prescribed macronutrient distribution in this group was 2 g·kg^−1^·d^−1^ of protein, 1 g·kg^−1^·d^−1^ of fat, and the balance of total calories from carbohydrates. Alternatively, the PER group progressively restricted caloric intake, accomplished by a reduction in carbohydrates, as follows: Energy intake for the first two weeks (weeks 1 and 2) amounted to 40 kcal·kg^−1^ with a similar macronutrient prescription to the SER group. During the next two weeks (weeks 3 and 4), total calorie intake was reduced to 35 kcal·kg^−1^ FFM. The following month continued in the same fashion, with calories reduced to 30 kcal·kg^−1^ FFM in the fifth and sixth weeks and then further reduced to 25 kcal·kg^−1^ FFM in the final two weeks (weeks 7 and 8). To help ensure compliance, participants recorded their daily macronutrient intake via a smartphone app (MyFitnessPal, LLC, CA, USA), which has been validated as viable tool for energy and macronutrient assessment (Teixeira et al. 2018). A sports nutritionist with experience in RT instructed participants on the proper use of the app and managed dietary consumption over the course of the study.

### Body composition

Body composition was measured seven days after menstruation in both the pre- and post-intervention periods to avoid potential confounding issues due to hormonal-induced fluctuations in extracellular water (Rosenfeld et al. 2008; Stachenfeld 2008). Total body and regional body composition were estimated via dual-energy X-ray absorptiometry (DXA). To eliminate the influence of fat-free adipose tissue (FFAT) and thus provide more accurate measures of changes in body composition, FFAT was eliminated on DXA-derived FFM (Heymsfield et al. 2002). The FFAT was estimated assuming that 85% of adipose tissue is fat using the equation (FM/0.85) × 0.15; the FFM-FFAT was then calculated and reported as we have done recently (Bonilla et al. 2021). This is recommended especially when large changes in body fat occur following an intervention (Abe et al. 2019).

Each subject was scanned by a certified technician, and computer algorithms distinguished bone and soft tissue, edge detection, and regional demarcations (software version APEX 5.6.0.7, Hologic Horizon A, Bedford, MA). For each scan, subjects wore sport clothes and were asked to remove all materials that could attenuate the X-ray beam (e.g., jewelry items, underwear containing wire, etc.). Calibration of the densitometer was checked daily against a standard calibration block supplied by the manufacturer. The abdominal region was delineated by an upper horizontal border located at half of the distance between acromion and external border of the iliac crests, a lower border determined by the external end of iliac crests and the lateral borders extending to the edge of the abdominal soft tissue. All trunk tissue within this standardized region was selected for analysis. To determine intertester reliability, two different observers manually selected the area for each subject with a coefficient of variation = 0.263%.

### Strength- and power-related assessments

For measurement of variables related to muscular strength and power, subjects were instructed to avoid vigorous exercise for 48 h before the tests in both the pre- and post-study periods. Participants performed a general warm-up consisting of light stretching and stationary cycling for 7–10 min prior to testing.

The countermovement jump (CMJ) test was performed on a jump mat (Smart Jump; Fusion Sport, Coopers Plains, Australia). Subjects initiated movement by reaching 90º of knee flexion while keeping their hands at the waist and their trunk erect, and then jumped vertically as high as possible. Instructions emphasized that the movement should be performed without interruption from the beginning to the end of the jump. Subjects performed 3–5 practice attempts for familiarization. Thereafter, two jumps were provided with a rest interval of 1 min between each trial; the highest value was used for analysis.

Pre- and post-study RM was assessed in the squat (SQ) and bench press (BP) performed on a Smith machine (Gervasport, Madrid, Spain). A specific warm-up set of the given exercise was performed for 12–15 repetitions at ~ 40% of subjects self-estimated 1-RM followed by two to three sets of two to three repetitions at a load corresponding to approximately 60–80% 1-RM. Participants then performed sets of one repetition of increasing weight for 1-RM determination. A 3- to 5-min rest interval was provided between each successive attempt. Participants were required to reach parallel in the 1-RM SQ; confirmation of squat depth was obtained by a research assistant positioned laterally to the subject to ensure accuracy. Successful 1-RM BP was achieved if the subject displayed a five-point body contact position (head, upper back, and buttocks firmly on the bench with both feet flat on the floor) and executed full-elbow extension. 1-RM SQ testing was conducted before 1-RM BP with a 7-min rest period separating tests. Participants then performed as many attempts as necessary until repetition failure, using the protocol described by McGuigan (2016). Bench placement was set by marking the floor with adhesive tape, to maintain the same placement for both measurements. All testing sessions were supervised by the research team to achieve a consensus for success on each trial.

### Statistical analysis

The results are expressed as mean ± standard deviation. The normality of the data was assessed with the Shapiro–Wilk test and the equality of variance with the Levene test. The comparison of the means of the variables (pretest vs. posttest) was performed with the paired t-test, and the effect size (ES) was calculated with Hedges' *g*, considering *a* ≤ 0.2 as small effect, 0.5 moderate effect, > 0.8 as large effect, and ≥ 1.30 as a very large effect (Rosenthal 1996). Likewise, to evaluate the effects and the comparison between the intervention groups, a general linear model (GLM) of repeated measures was performed, considering the Time (pre-test vs. post-test) and Group (PER vs SER) factors, and the Time × Group interaction. Additionally, between-group comparisons were made with estimation statistics (Ho et al. 2019). A *P*-value less than 0.05 (*P* < 0.05) was considered statistically significant for all tests. The analyses were performed with SPSS version 26 (IBM Corp., Armonk, NY, USA) and the effect size was computed with the R package Data Analysis using Bootstrap-Coupled Estimation (DABEST) v0.3.0 (Ho et al. 2019) within the R statistical computing environment version 4.0.0 (R Core Team 2020).

## Results

**Fig. 2:**
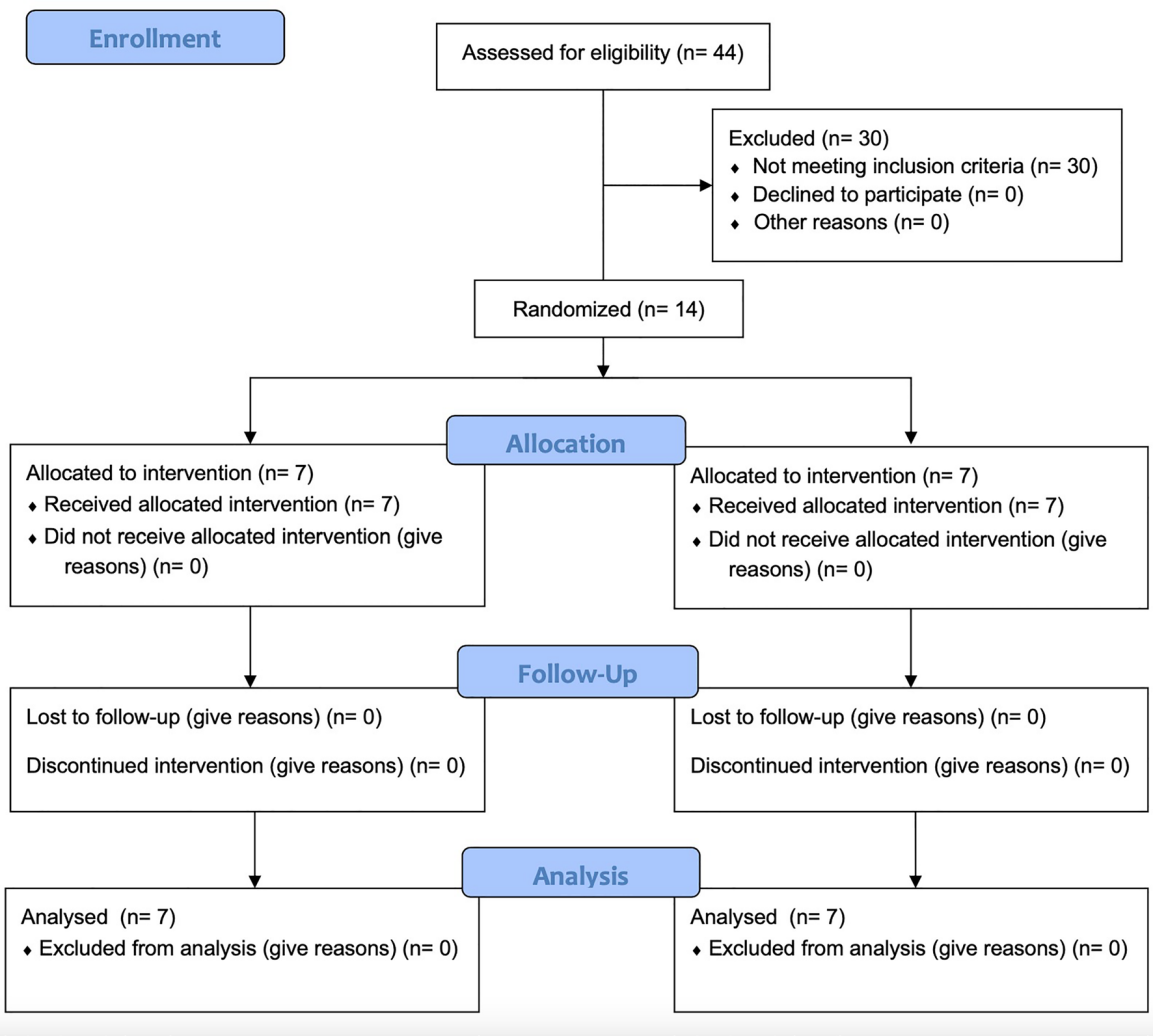
CONSORT diagram

Of the 44 women who participated in the immediately preceding study, only 14 met inclusion criteria and, thereafter, were assigned to either the PER (*n* = 7) or SER (*n* = 7) group. Figure 2 presents a flow diagram of subject enrollment as recommended by the Consolidated Standards of Reporting Trials (CONSORT).

Baseline analysis of the participants showed no between-group differences in any of the assessed variables (Table 1).

**Table 1:** Baseline of participants

	PER (*n* = 7)	SER (*n* = 7)	*P*
Age (y)	30.7 ± 3.2	28.3 ± 4.2	0.244
Stature (cm)	162.1 ± 8.4	167.7 ± 4.2	0.124
BM (kg)	59.4 ± 8.3	66.9 ± 8.6	0.146
BMI (kg/m^2^)	23.7 ± 3.0	23.9 ± 2.9	0.890
FM (kg)	16.8 ± 4.2	20.6 ± 6.0	0.193
FFM (kg)	42.6 ± 5.3	46.3 ± 4.3	0.183
FFAT (kg)	3.0 ± 0.7	3.6 ± 1.1	0.194
FFM-FFAT (kg)	39.7 ± 5.0	42.7 ± 4.0	0.243
BP (kg)	44.1 ± 7.6	42.6 ± 7.7	0.733
Squat (kg)	79.9 ± 9.6	73.4 ± 15.0	0.354
CMJ (cm)	27.2 ± 4.7	23.1 ± 4.9	0.136

*PER* progressive energy restriction, *SER* severe energy restriction, *BM* body mass, *BMI* body mass index, *FM* fat mass, *FFM* fat free mass, *FFAT* fat-free adipose tissue, *FFM-FFAT* fat free mas minus fat-free adipose tissue, *BP* bench press, *CMJ* countermovement jump

As planned, energy intake of the PER group was higher compared to SER in weeks 1–2 (*P* = 0.010). No differences were registered in weeks 3–4 and 5–6 (*P* = 0.167 and *P* = 0.120, respectively), while PER energy intake was lower compared to SER in weeks 7–8 (*P* = 0.003). Table 2 shows the estimated caloric intake from the nutrient intake record in both groups.

**Table 2:** Estimated nutrient and caloric intake through the intervention period

Groups	Weeks	Protein	CHO	Lipids	Caloric intake		P*
		(g/kg-FFM/d)	(g/kg-FFM/d)	(g/kg-FFM/d)	(kcal/kg-FFM/d)	(kcal/d)	
PER	1–2	2.6 ± 0.1	4.2 ± 0.2	1.4 ± 0.1	39.1 ± 0.7	1670.8 ± 220.3	0.010
	3–4	2.5 ± 0.1	3.4 ± 0.3	1.3 ± 0.1	35.0 ± 0.6	1492.4 ± 176.9	0.167
	5–6	2.4 ± 0.1	2.0 ± 0.5	1.3 ± 0.2	29.0 ± 0.6	1236.6 ± 151.4	0.120
	7–8	2.5 ± 0.2	1.4 ± 0.4	1.2 ± 0.2	25.9 ± 0.5	1101.8 ± 131.0	0.003
SER	1–8	2.6 ± 0.2	1.2 ± 0.5	1.6 ± 0.2	29.5 ± 0.6	1367.0 ± 140.0	–

*PER* progressive energy restriction, *SER* severe energy restriction, *CHO* Carbohydrates, *g/kg-FFM/d* grams per kilogram of fat-free mass, *kcal/kg-FFM/d* kilocalories per kilogram of fat-free mass per day, *kcal/d* kilocalories per day

*Compared to the caloric intake (kcal/d) of SER

**Fig. 3:**
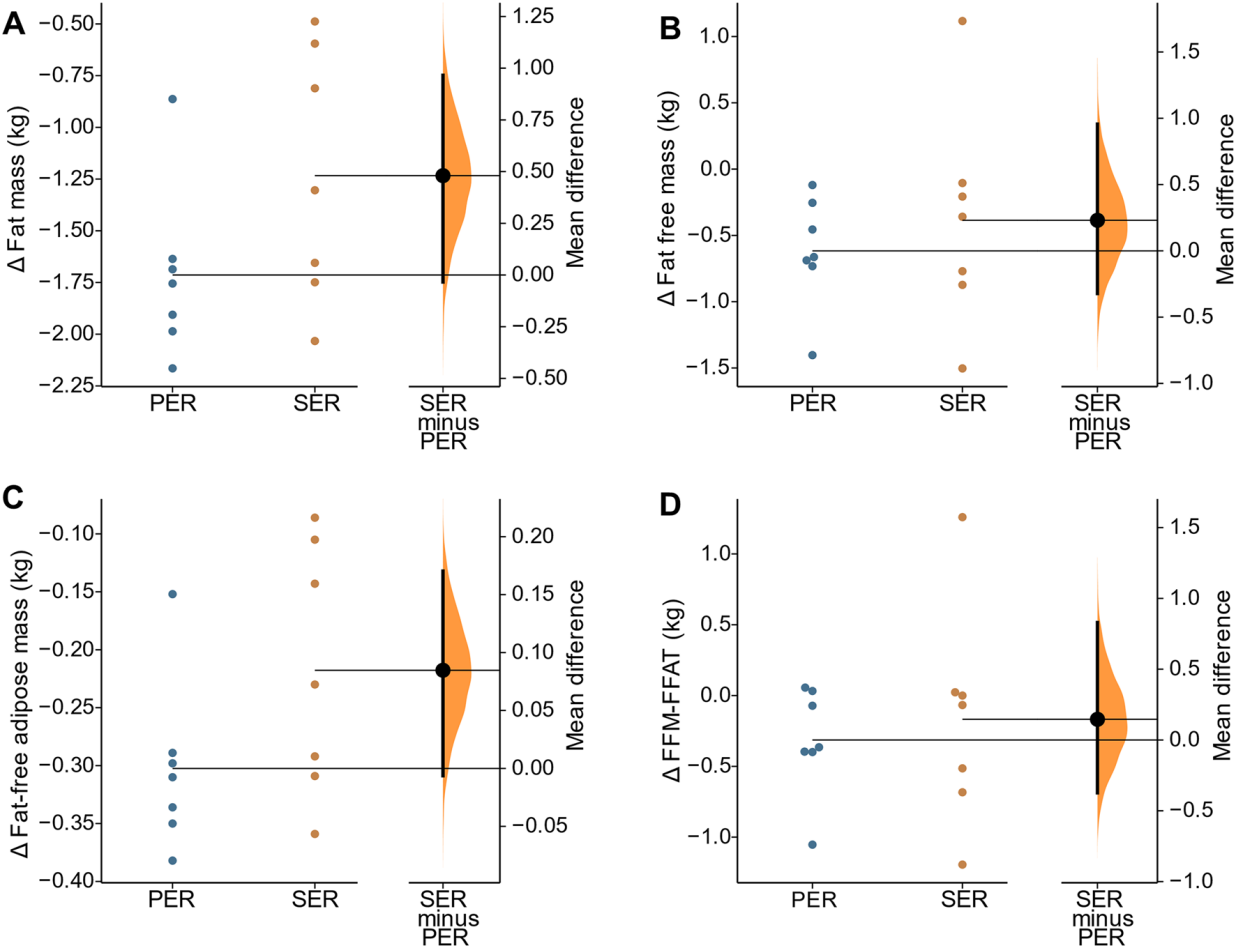
The difference between PER and SER in body composition. **A** Fat mass; **B** Fat free mass; **C** Fat-free adipose mass; **D** Fat free mas minus fat-free adipose tissue. The values presented are the post-intervention changes (post-test—pretest). In each figure, both groups are plotted on the left axes; the mean difference is plotted on a floating axes on the right as a bootstrap sampling distribution. The mean difference is depicted as a dot; the 95% confidence interval is indicated by the ends of the vertical error bar (Ho et al. 2019). PER, progressive energy restriction; SER, severe energy restriction

In regard to body composition, body mass showed a significant decrease with a small effect in PER (Δ = − 2.3 ± 0.7 kg; *P* < 0.001; ES = − 0.27) and SER (Δ = − 1.6 ± 1.2 kg; *P* = 0.013; ES = − 0.18). Similarly, FM was significantly reduced with a small effect in PER (Δ = − 1.7 ± 0.4 kg; *P* =  < 0.001; ES = − 0.39) and SER (Δ = − 1.2 ± 0.6 kg; *P* = 0.002; ES = − 0.20). FFM displayed a significant reduction with a small effect in PER (Δ = − 0.6 ± 0.4 kg; *P* = 0.008; ES = − 0.11); the reduction of FFM was not statistically significant in SER (Δ = − 0.4 ± 0.8 kg; *P* = 0.258; ES = − 0.08). However, when adjusting the FFM with the FFAT values (FFM–FFAT), no significant decrease was observed for the PER and SER groups (Δ = − 0.3 ± 0.1; *P* = 0.071; ES = − 0.06 and Δ = − 0.2 ± 0.1; *P* = 0.578, ES = − 0.04, respectively). No between-group differences were found on the change comparison (Fig. 3), nor in the GLM analysis for body composition variables (Table 2).

**Fig. 4:**
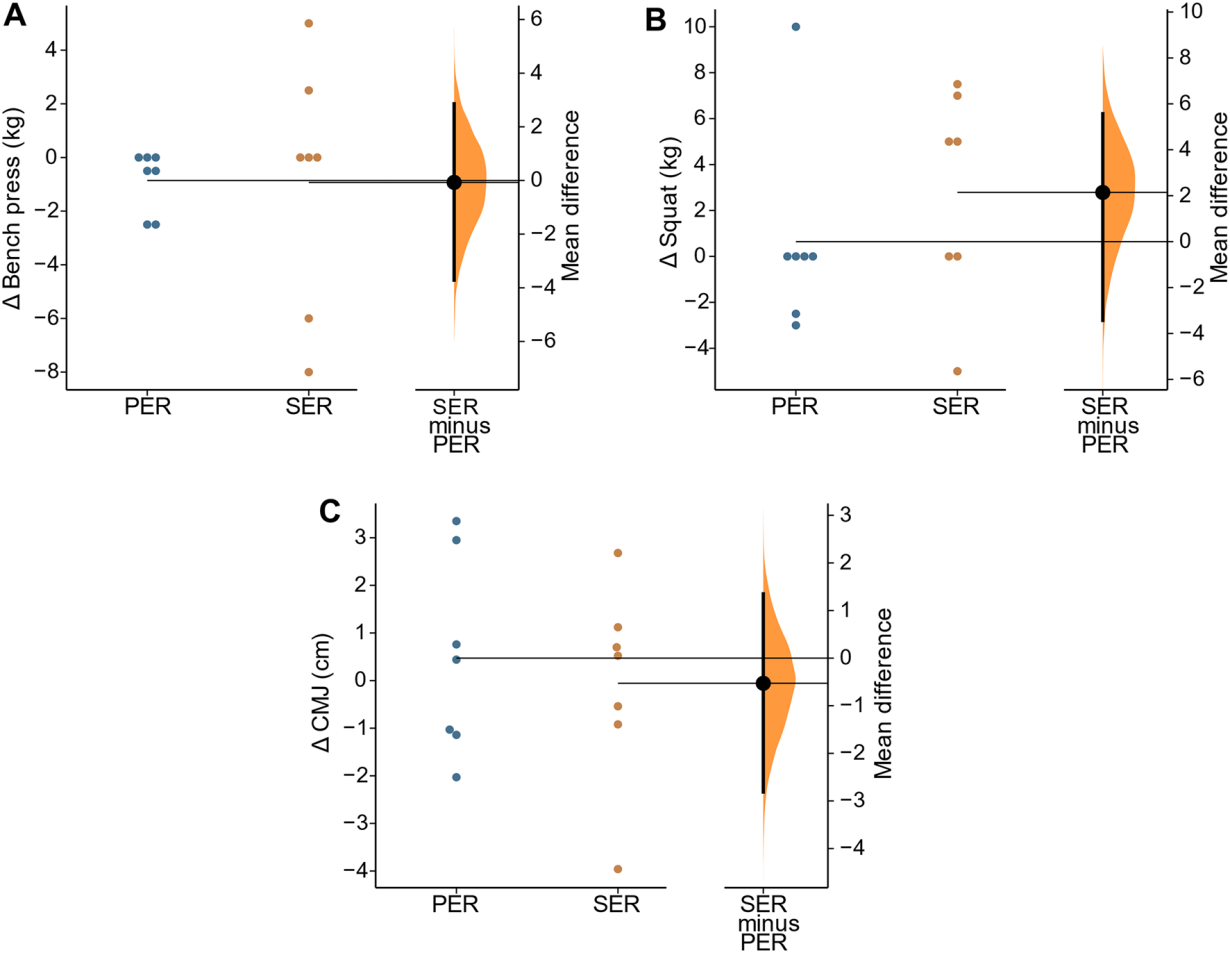
The difference between PER and SER in strength. **A** Bench press; **B** Squat; **C** CMJ. In each figure, both groups are plotted on the left axes; the mean difference is plotted on a floating axes on the right as a bootstrap sampling distribution. The mean difference is depicted as a dot; the 95% confidence interval is indicated by the ends of the vertical error bar (Ho et al. 2019). PER, progressive energy restriction; SER, severe energy restriction

In regard to the strength-related variables, no significant differences were found for PER or SER in the BP (Δ = − 0.9 ± 1.1 kg; *P* = 0.095; ES = − 0.11 and Δ = − 0.9 ± 4.6 kg; *P* = 0.610; ES = − 0.10, respectively), SQ (Δ = 0.6 ± 4.3 kg; *P* = 0.708; ES = 0.06 and Δ = 2.8 ± 4.6 kg; *P* = 0.159; ES = 0.17, respectively) or CMJ (Δ = 0.5 ± 2.1 cm; *P* = 0.568; ES = 0.10 y Δ = − 0.1 ± 2.1 cm; *P* = 0.944; ES = − 0.01, respectively). No between-group differences were found on the change comparison (Fig. 4), nor in the GLM analysis for the strength-related variables (Tables 2 and 3).

**Table 3:** Results of the study variables

Variable	Group	Before	After	Δ	*P*	ES	Time		Group		Time × Group	
							*P*	η^2^_p_	*P*	η^2^_p_	*P*	η^2^_p_
BM (kg)	PER	59.4 ± 8.3	57.1 ± 7.6	− 2.3 ± 0.7	0.0001	− 0.27	< 0.001	0.815	0.10	0.21	0.215	0.125
	SER	66.9 ± 8.6	65.3 ± 8.3	− 1.6 ± 1.2	0.013	− 0.18						
FM (kg)	PER	16.8 ± 4.2	15.0 ± 4.1	− 1.7 ± 0.4	< 0.001	− 0.39	< 0.001	0.903	0.16	0.158	0.111	0.197
	SER	20.6 ± 6.0	19.4 ± 5.7	− 1.2 ± 0.6	0.002	− 0.20						
FFM (kg)	PER	42.6 ± 5.3	42.0 ± 5.0	− 0.6 ± 0.4	0.008	− 0.11	0.01	0.41	0.17	0.154	0.518	0.036
	SER	46.3 ± 4.3	45.9 ± 4.4	− 0.4 ± 0.8	0.258	− 0.08						
FFAT (kg)	PER	3.0 ± 0.7	2.7 ± 0.7	− 0.3 ± 0.1	< 0.001	− 0.39	< 0.001	0.907	0.099	0.210	0.160	0.157
	SER	3.6 ± 1.1	3.4 ± 1.0	− 0.2 ± 0.1	0.002	− 0.20						
FFM-FFAT (kg)	PER	39.7 ± 5.0	39.4 ± 4.8	− 0.3 ± 0.4	0.071	− 0.06	0.160	0.157	0.665	0.016	0.230	0.118
	SER	42.7 ± 4.0	42.5 ± 4.2	− 0.2 ± 0.8	0.578	− 0.04						
1-RM_BP_ (kg)	PER	44.1 ± 7.6	43.2 ± 6.9	− 0.9 ± 1.1	0.095	− 0.11	0.336	0.077	0.73	0.01	0.969	< 0.001
	SER	42.6 ± 7.7	41.7 ± 9.6	− 0.9 ± 4.6	0.610	− 0.10						
1-RM_Squat_ (kg)	PER	79.9 ± 9.6	80.5 ± 9.5	0.6 ± 4.3	0.708	0.06	0.176	0.147	0.43	0.052	0.386	0.063
	SER	73.4 ± 15.0	76.1 ± 15.5	2.8 ± 4.6	0.159	0.17						
CMJ (cm)	PER	27.2 ± 4.7	27.6 ± 4.3	0.5 ± 2.1	0.568	0.10	0.72	0.01	0.11	0.196	0.642	0.019
	SER	23.1 ± 4.9	23.0 ± 5.5	− 0.1 ± 2.1	0.944	− 0.01						

*BM* body mass, *BMI* body mass index, *FM* fat mass, *FFM* fat free mass, *FFAT* fat-free adipose mass, *FFM-FFAT* fat free mas minus fat-free adipose tissue, *BP* bench press, *CMJ* countermovement jump, *PER* progressive energy restriction, *SER* severe energy restriction

## Discussion

The aim of this study was to compare two energy restriction protocols (PER vs SER) in combination with performance of a CT program on body composition and strength-related variables in trained women. Although our initial hypothesis was that the PER protocol would be more effective than SER for reducing FM and maintaining FFM and strength levels, no significant differences were observed between conditions. Consistent with our findings, Mero et al. (2010) reported statistically significant changes in FM reduction with no changes in lean body mass and bone mass in recreationally active, normal weight women when subjected to a deficit of 1000 or 500 kcal for 4 weeks (protein intake = 1.4–1.5 g·protein^−1^ per kilogram of BM). Alternatively, Garthe et al. (2011) found superior improvements in body composition and strength in a cohort of in 24 athletes (13 women) who targeted a slower vs faster weekly weight loss (0.7% vs 1.4% body mass per week) accompanied by 4 days/week of resistance training. Notably, our study is the first to compare a PER versus a SER in resistance-trained women and thus helps to fill current gaps in the literature. PER demonstrated a modestly greater magnitude of effect for changes in fat mass, which is of questionable practical significance.

Scrutiny of participants’ nutritional records in our study revealed that participants in the SER group did not consume the number of calories proposed (25 kcal·kg^−1^ FFM). This may help to explain the somewhat lower FM reduction (− 1.2 kg; ES =  − 0.20) compared to the PER group (− 1.7 kg; ES = − 0.39), although at the same time it indicates a greater adherence for PER. It should be noted that self-report nutritional records are prone to error (Schoeller 1995). Moreover, individual components of energy expenditure (i.e., resting metabolic rate and non-exercise activity thermogenesis) can influence energy requirements. Thus, explanations for relative differences in body composition measure between conditions remains speculative; it is possible that discrepancies simply are indicative of random noise of the measurement.

Regarding the possible differences between the sexes, Longland et al. (2016) demonstrated an increase in FFM following a 4-week RT program combined with a 40% caloric deficit (33 kcal·kg^−1^ FFM and 2.4 g·protein^−1^ per kg of BM) in untrained, overweight young men. Although our study did not show significant changes in FFM (corrected for FFAT), the biological differences between men and women must be taken into account in addition to the resistance training experience of our participants. It should also be noted that some of the participants in Longland et al. (2016) purportedly had previously engaged in resistance training, thus raising the possibility that increases in FFM were at least partially due to the “muscle memory” effect (Snijders et al. 2020).

Our study has several limitations that should be acknowledged. First, our sample size was relatively small, limiting statistical power; larger samples are needed to draw stronger practical inferences on the topic. Second, mood state was not assessed (Helms et al. 2015) to determine the lack of adherence in any of the groups. Third, the variation in energy intake among groups made it difficult to draw definitive conclusions. We did not measure resting energy expenditure which limited our discussion. Although no statistically significant differences between groups was detected on primary measures, we partially confirmed our initial hypothesis that PER might favor outcomes on body composition; nevertheless, additional more research is warranted to confirm this hypothesis. Finally, our study investigated one possible dietary comparison of energy restriction strategies; further research is needed to compare other nutritional strategies that have been shown to improve body composition in resistance-trained individuals (e.g., intermittent energy restriction such as intermittent fasting, diet refeeds or diet breaks) (Campbell et al. 2020; Escalante et al. 2020; Ashtary-Larky et al. 2021).

## Conclusions

PER and SER are effective strategies to reduce FM while maintaining FFM and strength levels in resistance-trained women undergoing an 8-week CT program. However, a progressive energy deficit appears to have a relatively higher magnitude of effect on FM reduction and might promote greater adherence in comparison to SER. Thus, our preliminary findings suggest that a PER may be considered a viable weight loss option in resistance-trained women.

## Data Availability

The datasets generated during and/or analysed during the current study are available from the corresponding author on reasonable request.

## Abbreviations

BM: Body mass
BMI: Body mass index
BP: Bench press
CMJ: Countermovement jump
CONSORT: Consolidated standards of reporting trials
CT: Concurrent training
DXA: Dual-energy X-ray absorptiometry
ES: Effect size
FM: Fat mass
FFM: Fat-free mass
FFAT: Fat-free adipose tissue
GLM: General linear model
RM: Repetition maximum
PER: Progressive energy restriction
SER: Severe energy restriction
SQ: Squat jump
VO_2_max: Maximal oxygen consumption
